# P-707. Trends in Respiratory Virus-associated Hospitalizations – National Healthcare Safety Network, United States, 2024 - 2025

**DOI:** 10.1093/ofid/ofaf695.919

**Published:** 2026-01-11

**Authors:** Catherine Lee, Jason A Fine, Matthew Heym, Andrea J Cool, Margaret Dudeck, Alison M Binder

**Affiliations:** Centers for Disease Control and Prevention, Atlanta, Georgia; Booz Allen Hamilton, Seattle, Washington; Booz Allen Hamilton, Seattle, Washington; Centers for Disease Control and Prevention (CDC), Atlanta, Georgia; Centers for Disease Control and Prevention, Atlanta, Georgia; Centers for Disease Control and Prevention, Atlanta, Georgia

## Abstract

**Background:**

Infections due to COVID-19, influenza, and respiratory syncytial virus (RSV) can lead to increase hospitalizations and strain the healthcare system during the winter respiratory season, when viruses are known to circulate at higher levels than during summer months. We assessed data on hospitalizations associated with COVID-19, flu, and RSV as reported to CDC’s National Healthcare Safety Network (NHSN) for the 2024-2025 respiratory season.

**Table 1:**
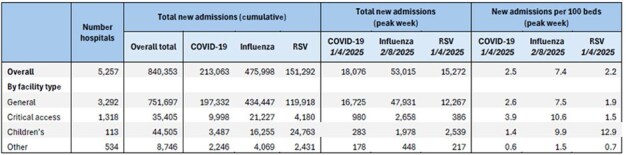
New hospital admissions for COVID-19, Influenza, and Respiratory Syncytial Virus (RSV) and rates per 100 inpatient beds nationally and by facility type, National Healthcare Safety Network (NHSN), November 2024 – April 2025

**Table 2:**
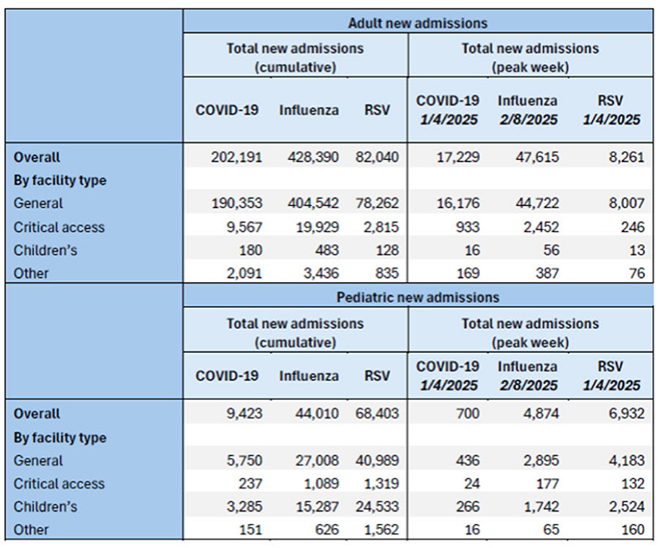
New hospital admissions for COVID-19, Influenza, and Respiratory Syncytial Virus (RSV) nationally and by facility type and age group, National Healthcare Safety Network (NHSN), November 2024 – April 2025

**Methods:**

Data collected from 5,257 acute, non-federal hospitals reporting to NHSN during November 2024 – April 2025 were included in the analysis. We evaluated and stratified the following metrics for each pathogen by facility type (general, critical access, and children’s) for each pathogen: cumulative admissions (overall and among adult and pediatric patients), peak admissions per 100 inpatient beds, and weekly mean percentage of patients hospitalized in the ICU.

**Table 3:**
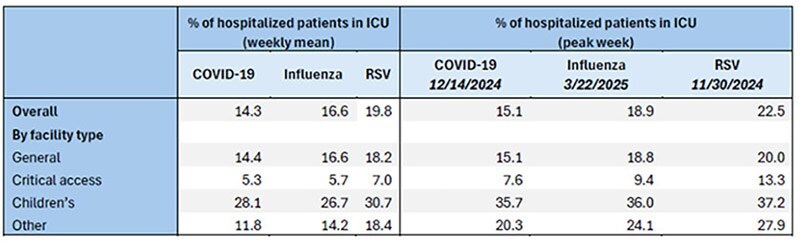
Percent of hospitalized patients with COVID-19, Influenza, and Respiratory Syncytial Virus (RSV) in Intensive Care Units (ICUs) nationally and by facility type, National Healthcare Safety Network (NHSN), November 2024 – April 2025

**Results:**

The highest cumulative admissions were for flu at 475,998, compared with 213,063 for COVID-19 and 151,292 for RSV (Table 1). Admissions related to COVID-19 and RSV peaked during the week ending 1/4/2025; admissions with flu peaked during week ending 2/8/2025. When examining admissions by age, the highest cumulative admissions among pediatric patients were for RSV at 68,403 compared with 44,010 for flu and 9,423 for COVID-19 (Table 2). RSV also demonstrated the highest mean percent of patients requiring ICU hospitalization at 20% compared with 17% for flu and 14% for COVID-19 (Table 3). When examining peaks in admissions by facility type, RSV had the greatest impact on 113 children’s hospitals (13 admissions per 100 beds). In contrast, flu and COVID-19 had the greatest impacts on 1,318 critical access hospitals (11 and 4 per 100 beds, respectively).

**Conclusion:**

The 2024-2025 season marked the first complete season in which all U.S. hospitals reported COVID-19, flu, and RSV hospitalization data to NHSN, enabling a comprehensive assessment of the burden of respiratory viruses on patients and hospitals. These data can be used in real-time during respiratory season for preparedness and response efforts, and future planning efforts, as the additive effect of COVID-19, flu, and RSV can adversely strain healthcare resources during peaks of respiratory season.

**Disclosures:**

All Authors: No reported disclosures

